# Coexistence of Median Arcuate Ligament Syndrome and Pancreatic Ductal Adenocarcinoma: A Case Report on Pancreaticoduodenectomy with Arterial Reconstruction

**DOI:** 10.70352/scrj.cr.25-0087

**Published:** 2025-06-07

**Authors:** Yuta Hiura, Tomoyuki Abe, Megumi Yamaguchi, Yusuke Sumi, Masatoshi Kochi, Ryuichi Hotta, Satoru Morita, Tsuyoshi Kobayashi, Hideki Ohdan, Kazuhiro Toyota

**Affiliations:** 1 Department of Gastroenterological Surgery, National Hospital Organization (NHO), Higashihiroshima Medical Center, Higashihiroshima, Hiroshima, Japan; 2 Department of Cardiothoracic Surgery, National Hospital Organization (NHO), Higashihiroshima Medical Center, Higashihiroshima, Hiroshima, Japan; 3 Department of Gastroenterological and Transplant Surgery, Graduate School of Biomedical and Health Sciences, Hiroshima University, Hiroshima, Hiroshima, Japan

**Keywords:** arterial reconstruction, celiac axis stenosis, pancreaticoduodenectomy

## Abstract

**INTRODUCTION:**

The celiac axis (CA) is usually dependent on blood supply from the superior mesenteric artery via the pancreatic arcade, particularly in cases of CA stenosis. During pancreaticoduodenectomy, excision of the gastroduodenal artery poses a significant risk of organ ischemia in the CA territory and may compromise anastomotic integrity. In cases of median arcuate ligament syndrome (MALS), blood flow typically improves after ligament transection. However, if atherosclerosis is present and chronic arterial compression is induced by the median arcuate ligament, stenting or revascularization may be required. Although revascularization is the most definitive technique, it raises concerns about anastomotic disruption due to postoperative pancreatic leakage. Considering these complexities, a thorough preoperative assessment of blood flow and the development of strategies to ensure adequate perfusion after resection are critical. Here, we encountered a patient with pancreatic cancer and MALS complicated by atherosclerosis.

**CASE PRESENTATION:**

A 76-year-old female patient with a history of acute appendicitis presented with generalized pruritus. Laboratory test results revealed significant elevations in her hepatobiliary enzymes and tumor markers. Imaging confirmed a 29-mm tumor in the pancreatic head and severe CA stenosis. Endoscopic ultrasonography and fine-needle aspiration confirmed pancreatic ductal adenocarcinoma. Due to severe CA stenosis and the inability to preserve the collateral vasculature, a multidisciplinary team decided to perform an abdominal aorta-to-splenic artery bypass using a saphenous vein graft. The surgery was successful, lasting 470 min with a blood loss of 700 mL. The patient was discharged on postoperative day 19 without complications and completed adjuvant chemotherapy. One year postoperatively, she remained recurrence-free with a patent graft and good hepatic artery flow.

**CONCLUSIONS:**

This report discusses a case of a successful pancreaticoduodenectomy with an abdominal aorta-to-splenic artery bypass without the complication of a pancreatic leak, thereby demonstrating the viability of the procedure for revascularization and reconstruction.

## Abbreviations

AA
: abdominal aorta
BMI
: body mass index
CA
: celiac axis
CEA
: carcinoembryonic antigen
CHA
: common hepatic artery
ERCP
: endoscopic retrograde cholangiopancreatography
FNA
: fine-needle aspiration
GDA
: gastroduodenal artery
MAL
: median arcuate ligament
PD
: pancreaticoduodenectomy
PDAC
: pancreatic ductal adenocarcinoma
PHA
: proper hepatic artery
SMA
: superior mesenteric artery
SpA
: splenic artery

## INTRODUCTION

CA stenosis or occlusion is detected in approximately 4%–10.5% of patients undergoing PD.^1,2)^ In cases of CA stenosis, the pancreaticoduodenal arcades form retrograde collateral pathways through the GDA from the SMA. PD involves division of the GDA, which is associated with a risk of ischemia of the liver and hepaticojejunal anastomosis.^3)^ Therefore, precise diagnosis of these hemodynamic abnormalities, either preoperatively or intraoperatively, is necessary, as well as consideration of methods to optimize blood supply to the liver. Stenting, MAL division, or revascularization are treatment options that are selected based on the pathological condition; however, no guidelines currently exist. The major causes of CA stenosis are MAL and atherosclerosis. Lateral angiography of the visceral aorta and computed tomography (CT) angiography (CTA) are the gold standards for the diagnosis of MAL syndrome (MALS), with compression images of the CA from the head side and changes similar to post-stenotic dilation.^4,5)^ Calcification around the abdominal axis is observed in many cases of CA stenosis due to atherosclerosis.^6)^ In this study, we encountered a patient with pancreatic cancer with MALS complicated by atherosclerosis.

## CASE PRESENTATION

**Fig. 1:**
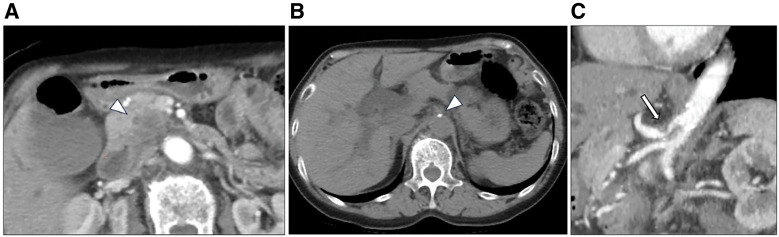
Enhanced abdominal CT (**A**) reveals a low-density tumor of the pancreatic head, 29 mm in diameter. Abdominal axial CT image (**B**) demonstrates calcification at the celiac axis. Sagittal CT image (**C**) revealing stenosis of the celiac artery with post-stenotic dilation and a hooked appearance. CT, computed tomography

**Fig. 2:**
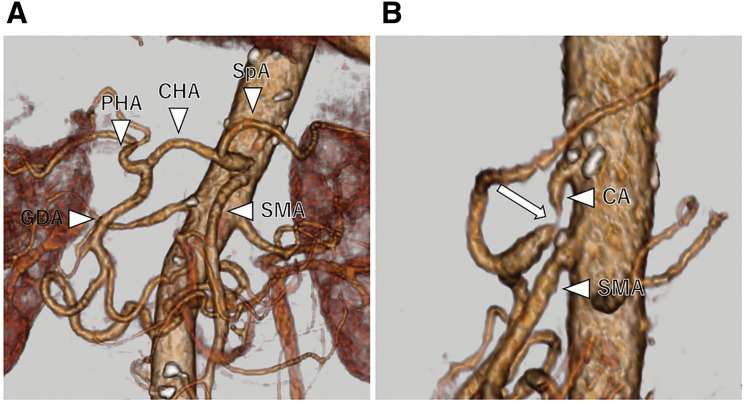
Preoperative 3D CT angiogram. (**A**) Development of the pancreatic arcade and dilatation of the GDA. (**B**) Severe stenosis of the CA as revealed by CT angiography. CA, celiac artery; CHA, common hepatic artery; CT, computed tomography; GDA, gastroduodenal artery; PHA, proper hepatic artery; SMA, superior mesenteric artery; SpA, splenic artery

**Fig. 3:**
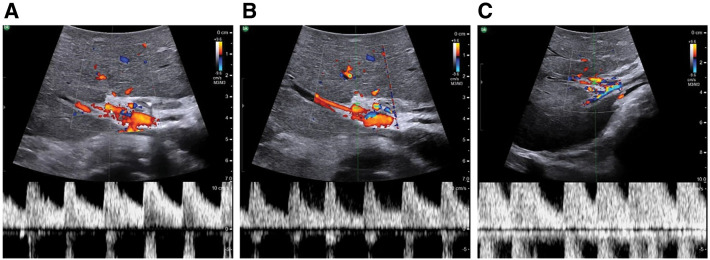
Doppler ultrasonography of hepatic artery flow. (**A**) Hepatic artery blood flow was well preserved before GDA clamping. (**B**) A decrease in hepatic artery flow was confirmed by GDA clamping. (**C**) Hepatic artery flow was fully restored following arterial reconstruction. GDA, gastroduodenal artery

**Fig. 4:**
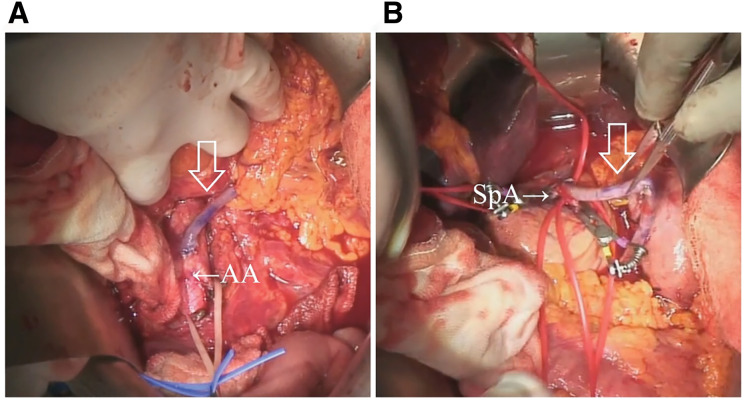
Intraoperative image. (**A**) The graft using the great saphenous vein was anastomosed to the aorta and reached the SpA through the transverse mesentery (arrow). (**B**) The graft was anastomosed to the SpA (arrow). AA, abdominal aorta; SpA, splenic artery

**Fig. 5:**
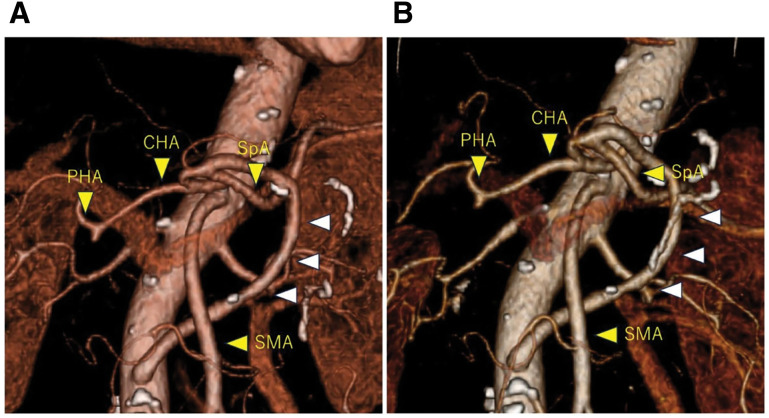
Postoperative 3D CT angiogram. (**A**) 3D CT image following pancreaticoduodenectomy with arterial reconstruction. (**B**) Follow-up 3D CT at 9 months postoperatively, showing no evidence of stenosis. CHA, common hepatic artery; CT, computed tomography; PHA, proper hepatic artery; SMA, superior mesenteric artery; SpA, splenic artery

A 76-year-old female patient presented to our hospital with generalized pruritus. Her medical history included the presence of acute appendicitis. The patient’s height, weight, and BMI were 153 cm, 45.2 kg, and 19.3 kg/m^2^, respectively. Initial laboratory results revealed markedly elevated hepatobiliary enzymes, including total bilirubin: 20.6 mg/dL); alanine aminotransferase: 632 U/L); aspartate aminotransferase: 459 U/L; alkaline phosphatase: 1157 U/L; and gamma-glutamyl transpeptidase: 1647 U/L. Tumor markers were also abnormally elevated, including Duke pancreatic monoclonal antigen type 2: 1825 U/mL; CEA: 4.4 ng/mL; and carbohydrate 19-9 antigens: ≤2.0 U/mL. Abdominal contrast-enhanced CT revealed a pancreatic head tumor with a maximum diameter of 29 mm and cephalic compression of the celiac artery with calcification (**Fig. 1**). CTA revealed CA stenosis and the development of a collateral artery from the SMA to the GDA (**Fig. 2**). ERCP identified that the distal bile duct was invaded by the pancreatic head tumor, and the upstream bile duct was diffusely dilated. Endoscopic ultrasound-guided FNA indicated that the tumor was an adenocarcinoma. Neoadjuvant chemotherapy was reduced to 1 cycle of gemcitabine and S-1 due to severe side effects. A multidisciplinary team involving radiology, cardiovascular surgery, and gastroenterological surgery was assembled to plan the treatment approach. MAL resection and stenting were considered infeasible due to calcification of the CA stenosis. Preservation of the collateral vasculature of the GDA was also considered oncologically inappropriate; therefore, bypass surgery was ultimately selected. An AA-to-SpA (AA-SpA) bypass was planned preoperatively. We expected that maintaining a distance between the AA-SpA bypass and the pancreaticojejunostomy would reduce the risk of anastomotic failure due to postoperative pancreatic fistula. During surgery, a GDA clamp test was performed. Attenuation of the hepatic artery pulse was observed, confirming decreased hepatic arterial blood flow on pulsed Doppler evaluation (**Fig. 3A** and **3B**). As expected preoperatively, revascularization was deemed necessary. AA-SpA reconstruction was performed using a 150-mm segment of the left saphenous vein graft before pancreatojejunal reconstruction. The anastomosis was completed using 7-0 Prolene in an end-to-side fashion over 20 min (**Fig. 4A** and **4B**). After the AA-SpA reconstruction, hepatic artery flow was fully restored, as confirmed by Doppler ultrasonography (**Fig. 3C**). Specifically, the operative time and blood loss were 470 min and 700 mL, respectively. The patient’s postoperative course was uneventful, and enhanced CT demonstrated that the AA-SpA bypass patency was preserved (**Fig. 5A**). She was started on 100 mg/day of aspirin on postoperative day 3 to prevent bypass obstruction and was discharged on postoperative day 19. Postoperative adjuvant chemotherapy with S-1 was completed. Efficient hepatic artery flow was observed with continued antiplatelet therapy, and the patient remained recurrence-free 1 year postoperatively (**Fig. 5B**).

## DISCUSSION

We report the successful arterial reconstruction with PD for pancreatic ductal adenocarcinoma (PDAC) in a patient with CA stenosis. Gaujoux et al.^7)^ reported that CT can detect significant CA stenosis with 96% sensitivity and determine its etiology with 92% accuracy. The key to accurately diagnosing CA stenosis lies in confirming the width of the GDA, which is significantly greater than that of the CHA and the normal arterial arcade. Another characteristic finding is the development of a collateral artery around the pancreatic head.^8)^ Therefore, examining not only the CA root but also the width of the GDA with the associated pancreatic head arcade is important to avoid a misdiagnosis of CA stenosis. Although the indication for revascularization in patients with CA stenosis is unknown, Sugae et al.^9)^ classified CA stenosis into 3 categories and reported that those with stenosis ranging from 80% to 100% are likely to require revascularization. Al-Saeedi et al.^10)^ also reported a higher probability of postoperative pancreatic fistula in patients with CA stenosis >80% when left untreated. Bull et al.^5)^ recommended the intraoperative GDA occlusion test as an effective tool for diagnosing celiac artery stenosis. The GDA clamp test revealing diminished hepatic arterial flow indicates the need for revascularization during PD.^11)^

The most frequent cause of CA stenosis was the MAL (46%), followed by atherosclerosis (42%).^12)^ Park et al.^13)^ reported that among the patients with calcified aortic plaque proven by CT, significant CA stenosis was found in 6.8% of cases. Furthermore, half of these CA stenoses were due to extrinsic compression by the MAL, which was identified as the most important etiology.

Various treatment options are available for treating CA stenosis. First, ligamentous dissection is useful for extrinsic compression caused by MALS.^7)^ The drawback of ligamentous dissection is the insufficiency of arterial blood flow due to chronic changes from the extrinsic compression. Despite these methods, MALS can develop acutely after PD, and is known as acute MALS (AMALS). Imai et al.^14)^ reported a case of celiac artery reocclusion after MAL division and postulated that preoperative catheter manipulation may have damaged the endothelial cells of the severely narrowed celiac artery. Although the pathogenesis of AMALS is unclear, a tight median arch ligament and lymph node dissection around the celiac artery are believed to be factors.^14,15)^ Second, successful endovascular treatment for CA stenosis after PD has been reported by Hanaki et al.^16)^ Considering the minimally invasive nature of IVR using covered balloon-expandable stents and its high success rate, stenting after MAL resection is recommended for treating CA stenosis.^17)^ However, the use of endovascular methods alone may be insufficient due to chronic vascular changes and external compression. Stent migration also remains a potential drawback. In cases of severe stenosis, ligament excision or stenting is inappropriate for insufficient patency; therefore, surgical treatment with preservation of the collateral vasculature or revascularization should be considered.^18)^ Third, the preservation of the collateral vessels carries the risk of positive resection margins. Revascularization is a superior alternative from an oncological perspective, particularly for malignant cases. Aneurysms are common in patients with peripancreatic collateral vessels associated with MALS^8)^; therefore, preservation of the collateral vessels leaves the possibility of aneurysm development or rupture.

In contrast, revascularization in PD raises concerns about postoperative bleeding due to pancreatic leakage, with reports of treatment failure in some cases.^19)^ Concerns also exist regarding the risk of thrombosis and graft occlusion; however, a report describes a long-distance bypass with an iliac artery-SpA using a 30 cm saphenous vein that was associated with successful short- and long-term outcomes.^20)^ Although graft-based revascularization is highly flexible and various routes have been used,^12)^ the safety of different routes has not been compared, and the method of choice remains unclear. Anastomosis of the SpA is relatively distant from the pancreatic pancreatojejunostomy and may be less susceptible to postoperative pancreatic leakage.

## CONCLUSIONS

Our case of successful PD with arterial reconstruction for PDAC in a patient with CA stenosis shows that arterial reconstruction is the most promising approach for achieving an oncological cure and ensuring hepatic artery flow. Furthermore, retrograde revascularization through the SpA may reduce the risk of ischemic accidents and prevent arterial anastomosis insufficiency caused by postoperative pancreatic fistulas.

## DECLARATIONS

### Funding

No funding body was involved in the design of the study, collection, analysis, and interpretation of data or in writing the manuscript.

### Authors’ contributions

HY and AT drafted the manuscript.

AT conceived the idea and developed the theory.

AT performed surgery.

All the authors discussed the results and approved the final version of the manuscript.

All authors have read and agreed on the final version of the manuscript.

### Availability of data and materials

Data sharing is not applicable to this article, as no datasets were generated or analyzed in the current study.

### Ethics approval and consent to participate

This work does not require ethical considerations or approval. Informed consent to participate in this study was obtained from the patient.

### Consent for publication

Written informed consent was obtained from the patient for the publication of this case report and the accompanying images.

### Competing interests

The authors declare that they have no competing interests.
